# Water surface tension modulates the swarming mechanics of *Bacillus subtilis*

**DOI:** 10.3389/fmicb.2015.01017

**Published:** 2015-09-24

**Authors:** Wan-Ju Ke, Yi-Huang Hsueh, Yu-Chieh Cheng, Chih-Ching Wu, Shih-Tung Liu

**Affiliations:** ^1^Department of Microbiology and Immunology, Chang Gung UniversityTaoyuan, Taiwan; ^2^Research Center for Bacterial Pathogenesis, Chang Gung UniversityTaoyuan, Taiwan; ^3^Graduate School of Biotechnology and Bioengineering, Yuan Ze UniversityTaoyuan, Taiwan; ^4^Department of Medical Biotechnology and Laboratory Science Proteomic Center, College of Medicine, Chang Gung UniversityTaoyuan, Taiwan; ^5^Department of Medical Research and Development, Chang Gung Memorial Hospital Chiayi BranchChiayi, Taiwan

**Keywords:** *Bacillus subtilis*, swarming, water surface tension, surfactin, quorum sensing

## Abstract

Many *Bacillus subtilis* strains swarm, often forming colonies with tendrils on agar medium. It is known that *B. subtilis* swarming requires flagella and a biosurfactant, surfactin. In this study, we find that water surface tension plays a role in swarming dynamics. *B. subtilis* colonies were found to contain water, and when a low amount of surfactin is produced, the water surface tension of the colony restricts expansion, causing bacterial density to rise. The increased density induces a quorum sensing response that leads to heightened production of surfactin, which then weakens water surface tension to allow colony expansion. When the barrier formed by water surface tension is breached at a specific location, a stream of bacteria swarms out of the colony to form a tendril. If a *B. subtilis* strain produces surfactin at levels that can substantially weaken the overall water surface tension of the colony, water floods the agar surface in a thin layer, within which bacteria swarm and migrate rapidly. This study sheds light on the role of water surface tension in regulating *B. subtilis* swarming, and provides insight into the mechanisms underlying swarming initiation and tendril formation.

## Introduction

Undomesticated *Bacillus subtilis* strains are known to swarm on agar surfaces (Kearns and Losick, 2003). Among these strains, *B. subtilis* 3610 has been studied extensively, as colonies formed by this strain expand rapidly at speeds of several centimeters per hour (Kearns and Losick, 2003). So far, the exact mechanisms underlying such rapid colony expansion are poorly understood. Earlier studies have established that flagella, which function as motors to produce a driving force for bacterial swarming (Wang et al., 2005; Kearns, 2013; Partridge and Harshey, 2013), increase in number and length during *B. subtilis* swarming (Kearns and Losick, 2003; Kearns, 2010). The bacteria also align and form rafts (Kearns and Losick, 2003; Kearns, 2010). In addition, *B. subtilis* swarming requires the production of a biosurfactant, surfactin (Kearns and Losick, 2003; Patrick and Kearns, 2009).

Cultivation of *B. subtilis* strain 3610 on 25 ml of Luria-Bertani (LB) medium containing 0.7% (LB-0.7) agar, under the experimental conditions established by Patrick and Kearns (2009), has been commonly used to investigate *B. subtilis* swarming, and the organism appears to swarm on the agar surface two-dimensionally as a monolayer in such conditions (Kearns and Losick, 2003). Following inoculation, *B. subtilis* 3610 goes through a lag period before swarming starts, during which bacteria are observed to be nonmotile (Kearns and Losick, 2003). The expression of *swrA*, which regulates swarming differentiation and flagellation (Calvio et al., 2005), as well as mutations in the Lon protease, have both been shown in earlier studies to shorten the swarm lag period (Kearns et al., 2004; Chen et al., 2009). Mukherjee et al. (2015) demonstrated that the Lon protease degrades SwrA when swarming motility inhibitor A (SmiA) is present during swimming, and further found that physical contact of bacteria with the solid agar surface inhibits SmiA-dependent proteolysis of SwrA to influence flagellation and swarming. The swarm lag period is also shortened when plates are inoculated with a high density of bacteria (Kearns and Losick, 2003), indicating that bacterial density can influence swarming. It is known that *B. subtilis* produces a 10-amino acid autoinducer peptide, ComX (Solomon et al., 1995), which accumulates as bacteria multiply. When ComX reaches threshold levels, a ComP-ComA two-component system is activated (Magnuson et al., 1994; Tortosa et al., 2001), and the phosphorylation of ComA then triggers transcription of the surfactin synthetase operon, *srfA*, leading to the synthesis of surfactin (Nakano and Zuber, 1991; Nakano et al., 1991a,b). However, it is currently unknown whether quorum sensing processes can shorten swarm lag and influence swarming.

An earlier study showed that a *B. subtilis* 3610 colony on LB medium containing 0.5% (LB-0.5) agar has a liquid-air interface (Be'er and Harshey, 2011), suggesting that *B. subtilis* colonies contain water and that bacterial cells are actually swarming in water. In fact, water is commonly observed in swarms formed by gram-negative bacteria, such as *Escherichia coli* and *Salmonella enterica* (Chen et al., 2007; Zhang et al., 2010; Wu et al., 2011; Ping et al., 2014). It is known that an *E. coli* swarm has water extending about 30 μm ahead of the edge of the swarm (Wu and Berg, 2012), and flagellar motion has been shown to induce fluid flows in *E. coli* swarms, pointing to the presence of substantial amounts of water (Wu et al., 2011). Fluid osmolarity within a swarm remains high, allowing water in the agar to be extracted, and a recent study found that an *E. coli* swarm has a high-osmolarity region at the edge and a low-osmolarity region within (Ping et al., 2014). It was suggested that the osmolytes are likely lipopolysaccharides (LPS), which can facilitate water extraction from the leading edge of a swarm (Ping et al., 2014).

Many *B. subtilis* strains are known to form colonies with tendrils during swarming, and the formation of these tendrils is influenced by culture conditions and nutrient availability (Kinsinger et al., 2003; Julkowska et al., 2004; Hamze et al., 2009; James et al., 2009); however, the exact mechanisms driving tendril formation remain unclear. In *Pseudomonas aeruginosa*, tendril formation is mediated by the actions of dirhamnolipids (di-RLs), which promote tendril formation and attract tendrils; and 3-(3-hydroxyalkanoyloxy) alkanoic acids (HAAs), which inhibit tendril formation and repel tendrils. The opposing forces generated by these two compounds are thought to cause tendrils to extend but avoid each other (Tremblay et al., 2007). In this study, we show that *B. subtilis* swarms in water, and further demonstrate that water surface tension plays a critical role in modulating many aspects of *B. subtilis* swarming behavior, such as swarming initiation and tendril formation.

## Materials and methods

### Strains and culture media

*B. subtilis* F29-3 is a wild-type strain (Chen et al., 1995). *B. subtilis* FK955 is a surfactin-synthesis mutant of F29-3, and contains a Tn*917ac1* transposon (Chang et al., 1994) inserted in *srfAB*. *B. subtilis* FW463, a flagellar mutant of F29-3, contains a Tn*917ac1* transposon inserted in *flgB*. *B. subtilis* 3610 and its flagellar mutant, DS1677, were kindly provided by Daniel B. Kearns (James et al., 2009). LB medium containing 0.4%, 0.6%, 0.7%, or 1.5% Oxoid agar (Thermo Scientific; LB-0.4, LB-0.6, LB-0.7, or LB-1.5) was used in swarming studies. Plates (9 cm), each of which contained 6, 8.5, or 25 ml of medium, were prepared. For *B. subtilis* F29-3 cultures, plates were prepared and sat at room temperature for 16 h, then dried again for another 15 min before inoculation. For inoculation, 1 μl of an overnight culture that had been grown in LB broth at 37°C was transferred to the center of a plate. The colony was dried for 10 min in a laminar flow hood and then incubated at 37°C. For *B. subtilis* 3610 cultures, LB-0.7 plates (25 ml) were prepared 16 h before experiments. After drying in a laminar flow hood for 25 min, plates were inoculated with 1 μl overnight culture or 10 μl of 10-fold concentrated log-phase *B. subtilis* 3610 cells, according to a method previously described by Patrick and Kearns (2009).

### Plasmid construction

A DNA fragment encoding the *gfp* gene with a ribosomal-binding sequence was amplified by PCR, using pEGFP-C1 (Clontech) as a template and the primers 5′-CGC GCTCGAGCCCCAGATCTGAGGAGGTTTAATTAATGAGCGCTAGCAAAGGAGA and 5′-CGCGTCTAGATTATTTGTATAGTTCATCCA. The fragment was inserted at the *Sma*I site in pHY300PLK (TaKaRa) to generate pRU-gfp. The *hag* promoter, from −200 to +92, was also amplified by PCR, using total DNA isolated from F29-3 and the primers 5′-CGCGTCTAGATGACCAAGTAAAAATTGGAA and 5′-CGC GGGATCCTGTTTTGTTCCTCCCTGAAT. The promoter was then inserted upstream of *gfp* at the *Xba*I-*Bam*HI sites in pRU-gfp to generate transcriptionally-fused pHag-gfp. The *srf* promoter region from −415 to +85 was amplified by PCR using primers 5′-CGC GTCTAGATGTAAATGGCTTTCAACAAT and 5′-CGC GGGATCCATTATCTTACCTCCCCTAAT, and then transcriptionally fused with the *gfp* gene by inserting the fragment at the *Xba*I-*Bam*HI sites in pRU-gfp, to obtain plasmid pSrf-gfp.

### Microscopy

Colony morphology was observed using a fluorescence stereomicroscope (Olympus SZX10). Bacterial motion was recorded using a CCD camera (JVC KY-F550). Bacterial motility was observed using a Zeiss Axioskop microscope at a magnification of 400 ×. To measure the height of colonies formed by GFP-expressing *B. subtilis* F29-3, bacteria were cultured on an LB-0.4 agar plate that contained 1/100th of its volume of 0.5 μm carboxylate-modified FluoSpheres beads (Invitrogen, Ex580/Em605). To measure the height of colonies formed by strain F29-3, a z stack in which the planes were separated by 10 μm was obtained with an upright laser-scanning confocal microscope (Leica, TCS-SP2). The last plane exhibiting red fluorescence outside the colony area was used to locate the surface of the agar plate. The distribution of green fluorescence was used to determine the height of the colony. This method was also used to measure the height of the colony formed by strain 3610, except that the bacteria were cultured on 25-ml LB-0.7 agar plates without red fluorescence beads, and z stacks with planes separated by 2 μm were acquired. In an experiment that examined the timing of the start of colony expansion, 1/100th of carboxylate-modified FluoSpheres beads were added to the inoculum to mark the location of inoculation. The expansion of the colony beyond the fluorescence zone was observed using an Olympus SZX10 stereomicroscope.

### Quantitative analysis of cells in a colony

*B. subtilis* F29-3 was cultured on LB-0.4 agar plates at 37°C. Single colonies were removed along with agar from a plate, using a surgical blade, and suspended in 10 ml PBS by vortexing. Cells in the suspension were serially diluted with PBS, and plated in triplicate on LB agar plates. Viable cell counts were conducted after 12 h of culturing at 37°C.

### Flagellar staining

Flagella of *B. subtilis* were stained using a silver stain method described elsewhere (West et al., 1977). In brief, 25 ml of aluminum potassium sulfate aqueous solution (saturated), 50 ml of 10% (v/v) tannic acid solution, and 5 ml of 5% ferric chloride solution were combined to prepare the mordant. The stain was prepared by slowly adding 2–4 ml of concentrated ammonium hydroxide to 90 ml of a 5% silver nitrate solution, to the point where the resulting brown precipitate just dissolves, after which more 5% silver nitrate solution was dripped into the solution until a faint cloudiness was observed. Both solutions were stored in the dark at 4°C. When staining, bacteria were picked up with an inoculating needle and smeared in distilled water on a slide, then covered with mordant for 4 min. After rinsing with distilled water, the stain was applied and slides were gently heated until steaming, whereupon staining continued for 4 min, and then slides were rinsed with distilled water and air-dried.

### LC-MS analysis of surfactin

Surfactin was purified from culture supernatants according to the acid-precipitation method described by Al-Ajlani et al. (2007). Briefly, *B. subtilis* was cultured in LB broth for 24 h, and after centrifugation to remove the cells, HCl was added to the culture supernatant to lower the pH to 2. The precipitate was collected by centrifugation and extracted with dichloromethane (J. T. Baker). The solvent was collected and removed under reduced pressure to give an off-white solid, which was dissolved in distilled water. NaOH was subsequently added to the solution to raise the pH to 8. The pH of the solution was adjusted to pH 2 again with concentrated HCl. After centrifugation, the supernatant was discarded and the pellet was dissolved in methanol. For surfactin quantification, each sample was diluted 2-fold with methanol, and subjected to fractionation by reverse-phase chromatography using the nanoACUITY UPLC column (Waters). Each sample (2 μl) was loaded onto an analytical C_18_ column (1.7 μm, 2.1 × 50 mm, Waters ACQUITY UPLC BEH C18) at a flow rate of 0.18 ml/min in 70% of solution A (0.1% formic acid) and 30% of solution B (99.9% acetonitrile containing 0.1% formic acid). Surfactin was eluted using a linear gradient of 30–95% solution B for 20 min, 95% solution B for 2 min, and 95–30% solution B for 1 min at a flow rate of 0.18 ml/min across the analytical column. The UV chromatogram at 220 nm was recorded. The surfactin standard (1 nanomole; Sigma-Aldrich) was quantified using HPLC-UV. Fractions correlating to surfactin from reverse-phase HPLC were confirmed using MALDI-TOF MS (Ultraflex TOF/TOF mass spectrometer, Bruker Daltonics). Peaks with *m/z* 1030, 1044, 1058, and 1072 correspond to the mass of [M + Na]^+^ ions of surfactin isoforms (Tang et al., 2010).

### Expression of GFP by swarms

*B. subtilis* cells were harvested along with agar from plates, and suspended in 10 ml PBS by vortexing. Following centrifugation at 14,000 × g for 5 min at 25°C to remove the agar, bacteria were resuspended in 400 μl PBS and homogenized by sonication on ice for 4 min at 100–200 watts, in cycles of 4-second sonication with 4-second pauses. The lysates were then centrifuged at 14,000 × g for 10 min at 4°C. Protein concentration was determined by the Bradford method (Bradford, 1976). Proteins (1 μg) in the lysates were analyzed by immunoblot analysis using anti-σ^A^ (kindly provided by Ban-Yang Chang) and anti-GFP (Sigma) antibodies, according to a method described elsewhere (Wang et al., 2011).

## Results

### Swarming behavior of *B. subtilis* F29-3

**Figure 1:**
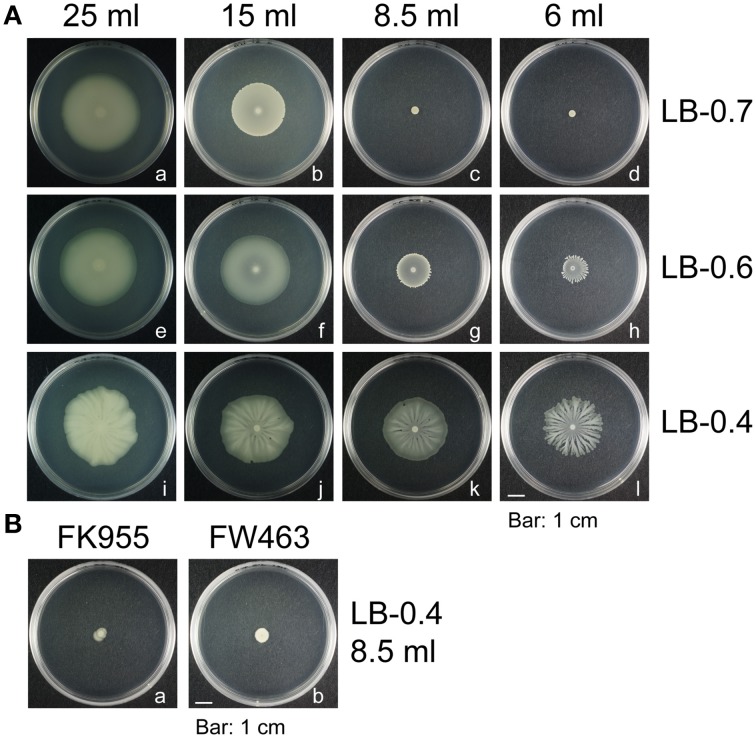
**Morphology of ***B. subtilis*** colonies**. **(A)** *B. subtilis* F29-3 was cultured on plates containing 6–25 ml LB medium containing 0.4% (LB-0.4), 0.6% (LB-0.6), or 0.7% (LB-0.7) agar. **(B)** FK955 (a surfactin-synthesis mutant of F29-3) **(a)**, and FW463 (a flagellar mutant of strain F29-3) **(b)** were cultured on 8.5-ml LB-0.4 agar plates. Plates were inoculated with 1 μl overnight culture and incubated at 37°C.

*B. subtilis* swarming is commonly studied using the 3610 strain, which swarms rapidly—colonies can expand and cover the entire surface of a 9-cm LB-0.7 plate in a few hours (Kearns and Losick, 2003). However, *B. subtilis* F29-3 swarms at slower speeds than 3610, and often develops colonies with tendrils. This study sought to elucidate the mechanisms underlying *B. subtilis* swarming by comparing the swarming behavior of these two strains. We inoculated LB plates with 1 μl F29-3 that had been grown in LB broth overnight. After culturing the bacteria at 37°C for 24 h, the strain was found to swarm on plates containing 25 ml of LB-0.7 agar, but did not swarm if the volume of medium was reduced to less than 15 ml (Figures 1Aa–d). The strain was also found to swarm on plates containing 8.5 ml or 6 ml of 0.6% (LB-0.6) agar, but at a reduced speed (Figures 1Ag,h). On LB plates containing 8.5 ml of 0.4% (LB-0.4) agar, the strain swarmed well, but developed tendrils when the agar volume was reduced to 6 ml (Figure 1Al). These results show that agar concentration and medium volume can affect the morphology of F29-3 swarms. We elected to use plates containing 8.5 ml of LB-0.4 agar for subsequent studies, as these had better light transmission in phase contrast microscopy.

### Genome and genes related to swarming in strain F29-3

*B. subtilis* F29-3 is a wild-type strain (Chen et al., 1995). The genome of this organism is 4,195,514 nucleotides long (accession number: AP014696), and contains genes that were shown to be involved in swarming in the 3610 strain (Table 1). After screening about 5000 mutant colonies that were generated using Tn*917ac1* (Chang et al., 1994), 12 of them were found to be unable to swarm on 8.5-ml LB-0.4 plates (Table 2). Among these mutants, FW463 had the transposon inserted in *flgB* (Table 2), and was defective in flagellar synthesis and swarming (Figure 1Bb; Supplementary Figure 1b). FK955 had a Tn*917ac1* insertion in *srfAB* (Table 2), and only swarmed on LB-0.4 agar after surfactin was applied to the medium prior to inoculation (Figure 1Ba; Supplementary Figures 1c,d). These results show that, as with the 3610 strain, F29-3 swarming requires surfactin and flagella.

**Table 1:** **Genes in F29-3 related to swarming**.

**Genes related to swarming**	**Length (bp)**	**Location (n.t.)^a^**
*swrC*	3162	726,621–729,782
*fla/che* operon	26,502	1,738,445–1,764,951
*swrA*	429	3,566,268–3,566,696
*yviB*; flagellar protein	420	3,585,707–3,585,288
*flgM*	267	3,585,208–3,584,942
*yviC*; flagellar protein	483	3,584,927–3,594,445
*flgK*	1524	3,584,426–3,582,903
*flgL*	897	3,582,893–3,581,997
*srf* operon	26,151	378,841–404,991

a *Accession number: AP014696*.

**Table 2:** **Swarming mutants of strain F29-3**.

**Mutant**	**Gene**	**Insertion site (n.t.)^a^**	**Function**
FK583	*kinD*	44	Histidine kinase of Spo0A
FK955	*srfAB*	3637	Surfactin syntherase
FK1816	*fliH*	468	Flagellar assembly protein H
FK1829	*comA*	11	Transcriptional factor of competence
FK1933	*srfAB*	3641	Surfactin synthetase
FK2116	*feuA*	3	Iron hydroxamate-binding protein
FK2197	*fusA*	1200	Elongation factor G
FK2230	*sinI*	158	Antagonist of SinR
FK2259	*flhA*	579	Flagellar biosynthesis protein
FW463	*flgB*	74	Flagellar basal-body rod protein
FW502	*fliK*	326	Flagellar hook-length control protein
FW506	*fliE*	133	Flagellar hook-basal body protein

a *Tn917ac1 insertion; from translation initiation codon*.

### Synthesis of surfactin by strains F29-3 and 3610

**Figure 2:**
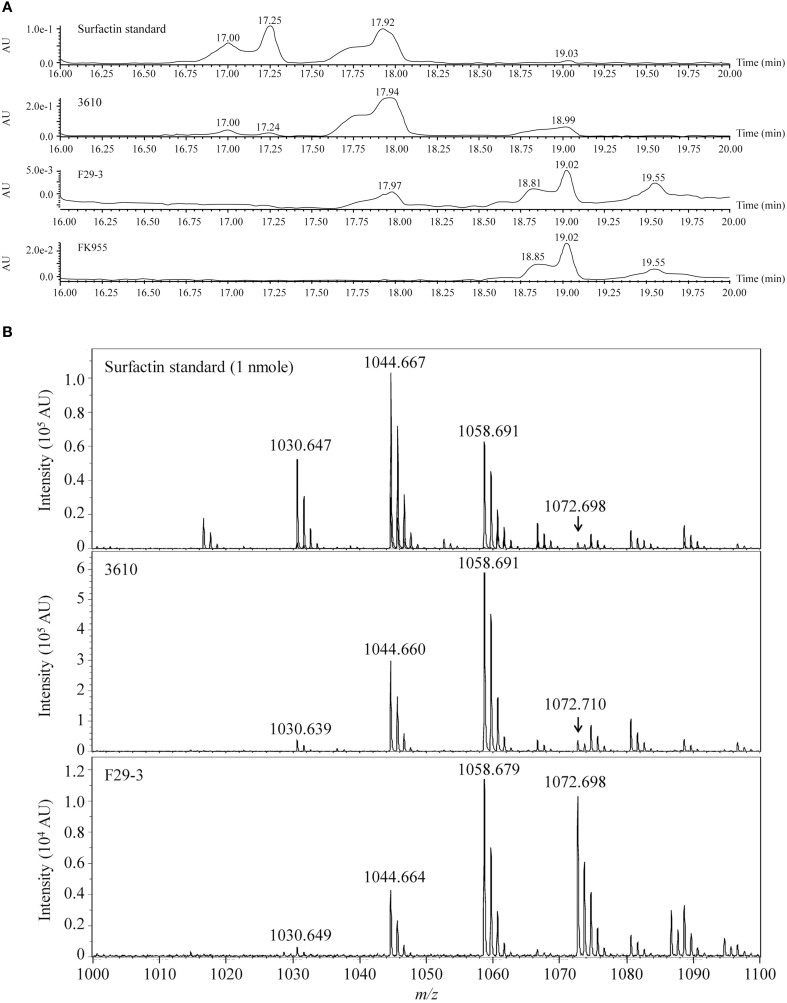
**LC-MS of surfactin purified from strains 3610, F29-3, and FK955**. **(A)** Strain 3610 was cultured in 50-ml LB broth; strains F29-3 and FK995 were cultured in 1 liter LB broth for 16 h. Surfactin in the culture supernatants was purified and analyzed by HPLC. Pure surfactin was used as a standard. **(B)** The HPLC peaks with retention times of 17.94 (strain 3610) and 17.97 (strain F29-3) were analyzed by MALDI-TOF mass spectrometry. The peaks with m/z 1030, 1044, 1058, and 1072, which correspond to the mass of [M + Na]+ ions of surfactin isoforms, are indicated. AU, Absorbance Unit.

We analyzed 1 nanomole of surfactin (Sigma-Aldrich) by liquid chromatography to serve as a standard, and found that the pure compound yielded three peaks with retention times of 17.00, 17.25, and 17.92 min (Figure 2A). We also cultured strain 3610 in 50 ml LB broth for 16 h, precipitated surfactin from the culture supernatant under acidic (pH 2) conditions, and then extracted surfactin using dichloromethane (Al-Ajlani et al., 2007). Chromatographic analysis of the extract similarly revealed peaks at 17.00, 17.24, and 17.94 min (Figure 2A). We then purified surfactin from 1 liter of F29-3 culture supernatant, and liquid chromatography analysis revealed one peak with a retention time of 17.97 min (Figure 2A). A parallel experiment showed that a surfactin-synthesis mutant of F29-3, FK955, did not produce such a peak (Figure 2A). Surfactin concentration in the 3610 culture medium was estimated to be 5.6 μM, but only 3.2 × 10^−4^ μM in the F29-3 culture medium, which is 17,519-fold less than that produced by strain 3610 (Table 3). Analysis of the chromatographic peaks at the retention time of 17.9 min by MALDI-TOF mass spectrometry revealed peaks with *m/z* 1030, 1044, 1058, and 1072 (Figure 2B), which corresponds to the mass of [M + Na]^+^ ions of surfactin isoforms (Tang et al., 2010) and confirming that the chromatographic peaks are those of surfactin. In addition to surfactin, strain F29-3 is also known to produce fengycin, but fengycin appears to be unrelated to swarming (Luo et al., 2014).

**Table 3:** **Surfactin expression of ***B. subtilis*** 3610, F29-3, and FK955**.

**Sample**	**HPLC Retention Time (min)**	**Peak Area**	**Surfactin Concentration**	
			**Observed (nmole)**	**Concentration (mM)**
Surfactin 1 nmole	17.92	26571	1	–
3610^a^	17.94	74468	2.803^a^	5.606
F29-3^b^	17.98	413	0.016^b^	3.2 x 10^−4^
FK955^b^	17.9 –18.0	0	0.000^b^	0.000

a *Purified from 50 ml LB culture; 1/100 of purified sample was used for LC-MS*.

b *Purified from 1000 ml LB culture; 1/20 of purified sample was used for LC-MS*.

### Swarming of *B. subtilis* F29-3

**Figure 3:**
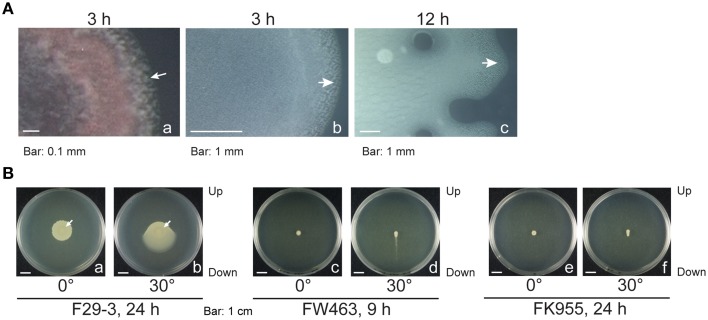
**Presence of water in ***B. subtilis*** F29-3 colonies**. **(A)** An LB-0.4 plate was inoculated with bacteria that were mixed with red fluorescence beads and cultured for 3 h. Images of the bacterial colony and red fluorescence were merged to show the expansion of the colony from the inoculation site **(a)**. *B. subtilis* F29-3 colonies without the red fluorescence beads were cultured for 3 h **(b)** and 12 h **(c)**, and observed under a stereomicroscope. White arrows indicate motile bacterial packs. **(B)** *B. subtilis* F29-3 colonies were cultured for 24 h on LB-0.4 agar plates that were laid flat **(a)** or tilted 30° **(b)**. *B. subtilis* FW463 and FK955 were cultured for 9 h and 24 h, respectively, on LB-0.4 agar plates that were laid flat **(c,e)** or tilted 30° **(d,f)**. Arrows indicate inoculation sites.

We inoculated LB-0.4 plates with F29-3 that had been mixed with 0.5 μm red carboxylate-modified FluoSpheres beads (Invitrogen), which were used to mark the site of inoculation, and observed the colony hourly for a period of 12 h using a fluorescence stereomicroscope. We found that the colony started to expand beyond the red fluorescent region at Hour 3 (Figure 3Aa), indicating that bacteria had exited the swarm lag period. Similar swarming behavior was observed when fluorescent beads were not added (Figure 3Ab), thereby demonstrating that the beads did not influence swarming. We also observed that bacteria aggregated and moved three-dimensionally in all directions when colony expansion started after Hour 3 (Supplementary Movie 1), suggesting the presence of fluid within the colony. At Hour 12, the size of the colony reached a diameter of about 1 cm; cells close to the 1 mm region at the edge of colony remained motile but those inside the colony became nonmotile (Figure 3Ac; Supplementary Movie 2). To verify the presence of water within the colonies, we cultured the bacteria on an LB-0.4 agar plate that was tilted 30°. After 24 h of culturing, the bacterial population traveled downward 2.4 cm, but only 1.1 cm upward (Figure 3Bb). In a parallel experiment, colonies expanded evenly from the center if a plate was not tilted (Figure 3Ba). This indicates that the gravitational pull exerted upon water in the bacterial colonies induces bacteria to swarm in the direction of gravity. We also observed that FK955 swarmed only slightly in the direction of gravity after 24 h of culturing (Figure 3Bf), suggesting that surfactin is needed to weaken water surface tension to facilitate swarming. A similar experiment was conducted using a flagellar mutant of F29-3, FW463. After 9 h of culturing on a tilted plate, water was found to flow downward out of the colony (Figure 3Bd). Instead of flowing downward radially as in F29-3 colonies (Figure 3Bb), FW463 formed comet-shaped colonies, with sparse bacterial growth observed in the comet tail regions (Figure 3Bd). These results indicate the presence of water within the bacterial colonies, and show that bacteria moved out of the colony with the flow of water in the direction of gravity.

### Shape and height of *B. subtilis* colonies

**Figure 4:**
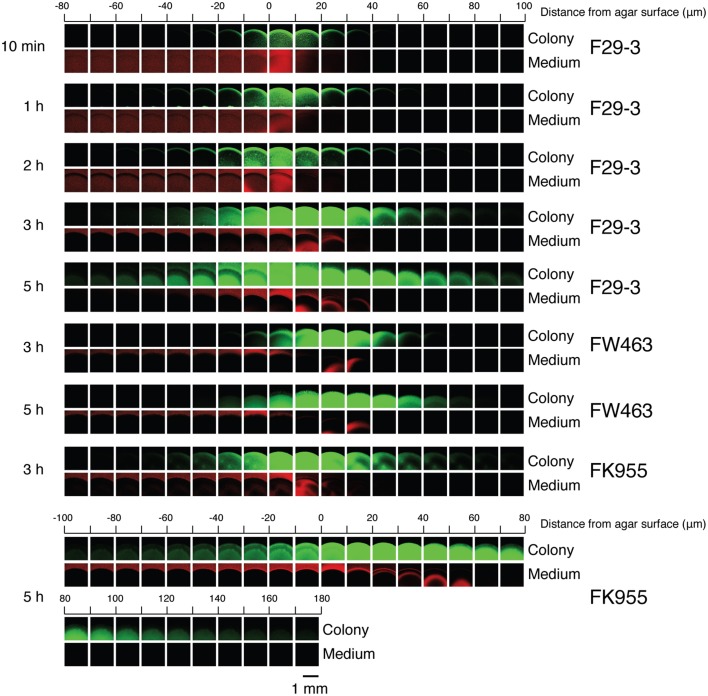
**Height and shape of colonies**. Z stacks, with planes separated by 10 μm, of colonies formed by *B. subtilis* F29-3(pHag-gfp), *B. subtilis* FW463(pHag-gfp) and *B. subtilis* FK955(pHag-gfp) were acquired using a confocal laser-scanning microscope. Bacteria were cultured on 8.5-ml LB-0.4 agar plates containing red carboxylate-modified FluoSpheres beads (Invitrogen). The last plane with red fluorescence outside the green colony area indicates the agar surface.

This study examined the morphology and measured the height of GFP-expressing *B. subtilis* F29-3(pHag-gfp) colonies, using a confocal laser-scanning microscope. Z stacks of the colonies were acquired, with planes separated by 10 μm, and the results revealed that colonies were initially flat at the center after inoculation, but rose to 100 μm with 5 h of culturing (Figure 4), which is about 30 times the length of a single bacterial cell. Additionally, the colony surface had a convex shape, with bacteria-occupied areas decreasing gradually from the lower to the upper planes (Figure 4). Colonies formed by a flagellar mutant, FW463(pHag-gfp), were about 60 μm in height at Hour 3 post-inoculation (Figure 4). At Hour 5, the height of the colony increased to about 80 μm. The dramatic rise in colony height prior to swarming suggests that bacteria are extracting water from the agar medium to fill their colonies. By contrast, colonies formed by a surfactin-synthesis mutant, FK955(pHag-gfp), also had a convex shape, reaching a height of about 160 μm after 5 h of culturing (Figure 4). The results from FK955(pHag-gfp) suggest that *B. subtilis* colonies contain water and are unable to weaken water surface tension in the absence of surfactin, leading to a substantial rise in colony height as bacterial densities increase and more water is taken up into the colony.

### Water surface tension plays a key role in F29-3 swarming

**Figure 5:**
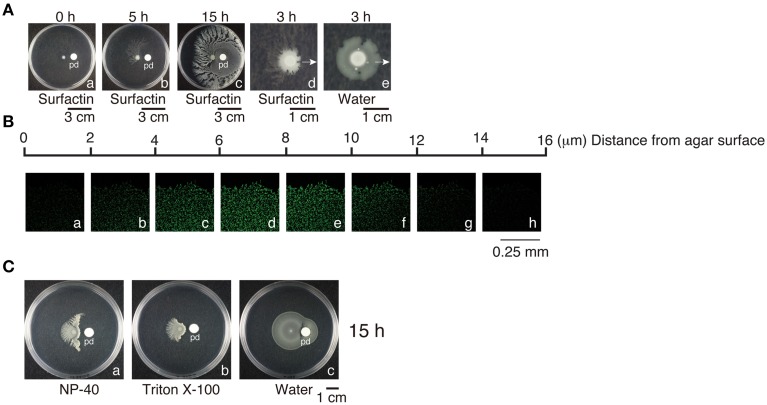
**Water surface tension and swarming**. **(A)** Paper discs (pd), to which surfactin (100 μl, 0.025%) **(a–d)** or 100 μl water **(e)** had been applied, were placed 1.5 cm away from the center of *B. subtilis* F29-3 colonies that had been cultured for 6 h on 8.5-ml LB-0.4 agar plates. Colonies were photographed at selected time points within 0–15 h after applying surfactin. Arrows indicate the direction of paper disc. **(B)** A paper disc with surfactin was placed 1.5 cm from the center of a 6-h *B. subtilis* F29-3(pHag-gfp) colony. The bacteria were cultured for an additional 2 h. Z stacks of the edge of the colony facing the paper disc, with planes separated by 2 μm, were acquired using a confocal laser-scanning microscope. **(C)** Filter paper discs, to which NP-40 **(a)**, Triton X-100 **(b)** (100 μl, 0.025%), or 100 μl of water **(c)** were respectively applied, were placed 1.5 cm from the center of *B. subtilis* F29-3 colonies cultured for 6 h. Plates were incubated for 15 h.

Since *B. subtilis* F29-3 colonies contain water, and water surface tension may restrict colony expansion, we sought to ascertain whether water surface tension plays any role in swarming. Accordingly, we applied 100 μl of 0.025% surfactin to a paper disc that was placed 1.5 cm away from the center of an F29-3 colony that had been cultured for 6 h (Figure 5A). This created a surfactin gradient in the medium, with surfactin concentration being higher at the side of the colony facing the paper disc than that of the distal side. The presence of surfactin, which is a powerful surfactant, reduced the water surface tension of the colony at the side closest to the paper disc, causing the water to spread; as a result, the height of the colony was reduced from 100 μm to 14 μm at 3 h after applying surfactin (Figures 5Ba–h). The bacteria in this thin water layer formed rafts and swarmed (Supplementary Movie 3), and passed the paper disc in about 2 h. After 15 h of culturing, a thin layer of bacteria grew and surrounded the paper disc (Figure 5Ac), with overall morphology similar to *B. subtilis* 3610 colonies post-swarming (Kearns and Losick, 2003). Meanwhile, at the distal side of the colony, where surfactin concentration was relatively low, tendrils emerged as early as 3–5 h after applying surfactin (Figures 5Ab,d). The application of detergents such as NP-40 or Triton X-100 induced similar effects (Figures 5Ca,b); however, a parallel experiment showed that bacterial colonies did not develop tendrils if water was applied to the disc instead (Figure 5Ae,Cc), demonstrating that weakening water surface tension promotes tendril formation.

### Water in colonies formed by strain 3610

**Figure 6:**
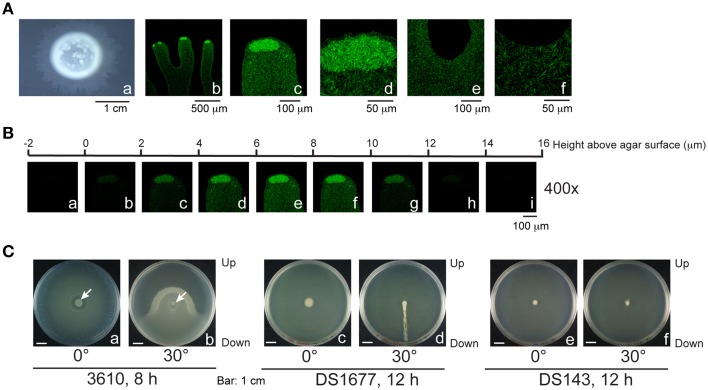
**Migration of ***B. subtilis*** 3610 in flowing water**. **(A) (a)** *B. subtilis* 3610 colonies cultured on 25-ml LB-0.7 agar plates for 3 h. **(b–f)** Compressed confocal images from the tendril **(b)**, tendril tips **(c,d)**, and the base of two tendrils **(e,f)** of *B. subtilis* 3610(pHag-gfp) colonies are shown. **(B)** Z stack of the edge of a *B. subtilis* 3610(pHag-gfp) colony. **(C)** *B. subtilis* 3610 **(a,b)** and *B. subtilis* DS1677 (a flagellar mutant) **(c,d)** and DS143 (a surfactin-synthesis mutant) **(e,f)** colonies cultured for 24 h on LB-0.7 agar plates that were laid flat **(a,c,e)** or tilted 30° **(b,d,f)**.

**Figure 7:**
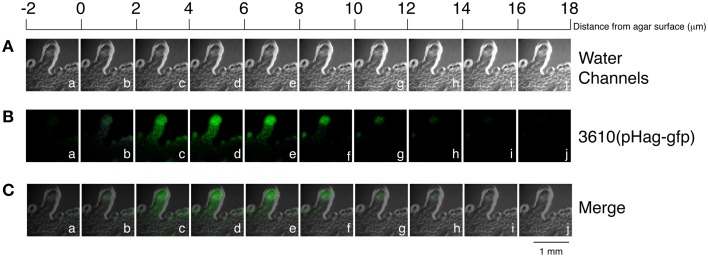
**Height of the edge of a colony formed by ***B. subtilis*** 3610(pHag-gfp)**. A z stack, with planes separated by 2 μm, was acquired from the edges of a *B. subtilis* 3610(pHag-gfp) colony that had been cultured for 2 h on a 25-ml LB-0.7 plate (green) **(B)**. A z stack was also generated using a visible light to show the edge of the colony (gray) **(B)**. Merged images of **(A)** and **(B)** are shown in **(C)**.

We further examined the swarming behavior of *B. subtilis* 3610, a strain that swarms rapidly on LB agar. This strain was shown to swarm on the surface of LB-0.7 agar as a monolayer (Kearns and Losick, 2003). Be'er and Harshey (2011) also demonstrated that *B. subtilis* 3610 on LB-0.5 agar swarms between the agar surface and a liquid-air interface, suggesting that the swarm contains water. *B. subtilis* 3610 swarming is commonly studied on LB-0.7 agar (Kearns and Losick, 2003; Patrick and Kearns, 2009), but the presence of water in colonies formed on this type of agar was not observed by phase contrast microscopy. Since the presence of water in such swarms has not been documented previously, we subsequently sought to examine this issue. Plates containing 25 ml of LB-0.7 agar were inoculated with 1 μl overnight culture. After 3 h of culturing, a thin layer of bacteria with a lobate edge emerged from the colony (Figure 6Aa). We observed that the central region of the colony was densely packed with both nonmotile and motile bacteria, but a few motile bacteria moved out of the center toward the edge of the colony. Measurements of the edge areas by confocal microscopy revealed the overall height to be about 12–14 μm, with the majority of bacteria floating in the region between 4 μm and 10 μm (Figure 6B). Tilting the plate caused bacteria to swarm in the direction of gravity (Figure 6Cb), indicating that the colony contains water and that bacteria swarm upward against gravity inefficiently in the absence of water flows. Additionally, when cultured on a tilted plate, colonies of DS1677 (a flagellar mutant of 3610), did not expand or demonstrate swarming behavior, but a trail of bacteria leading toward the bottom of the plate emerged from the colony after 12 h of culturing (Figure 6Cd). We also found the surfactin-synthesis mutant, DS143, did not swarm toward gravity (Figures 6Ce,f), indicating that surfactin is required for swarming. Colonies formed by 3610 had numerous transitory tendrils at the edges, with motile bacteria accumulating and swirling at the tips (Figures 6Ab–d). Within these tendrils, bacteria originating from the central region of the colony were sparse (Figures 6Ae,f), and appeared as a monolayer under a phase contrast microscope. Examination of a particular tendril found that it extended at speeds of 2.67 μm/s at the tip and 1.07 μm/s at the base where two tendrils are joined, with similar extension profiles observed in other tendrils surveyed. These results suggest that tendrils gradually merge at the base to form a colony without branching, while expanding very little in a sideways direction. The lack of sideways expansion suggests that these tendrils are actually water channels, as bacteria are unlikely to move steadily in a set direction. The accumulation and swirling of bacteria at channel tips (Figures 6Ab–d) suggest that bacteria actively migrate along channels and are not simply propelled by water flow, but are stopped by water surface tension at the tips. We also cultured 10-fold concentrated log-phase cells on 25-ml LB-0.7 agar, according to the methods described by Patrick and Kearns (2009), and found that bacteria similarly formed 14-μm water channels, with accumulation seen at channel tips (Figure 7).

### Quorum sensing and surfactin synthesis by F29-3 and 3610 swarms

**Figure 8:**
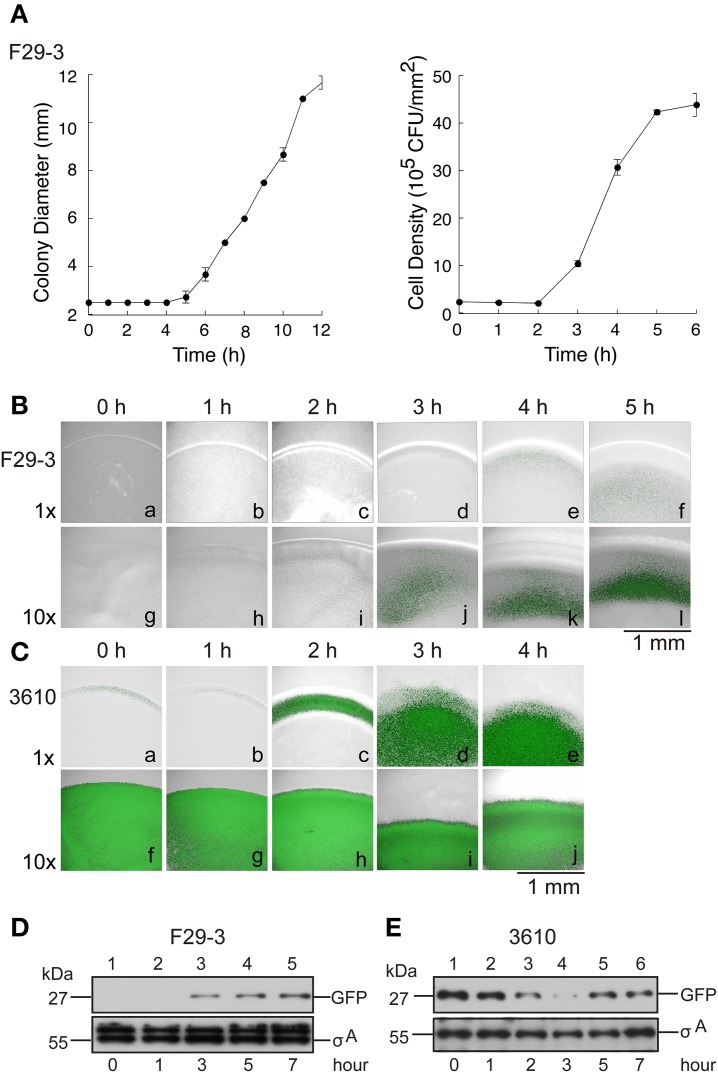
**Quorum sensing and its role in swarming**. **(A)** LB-0.4 plates were inoculated with *B. subtilis* F29-3. The size of the colonies was measured with a ruler. Cell numbers were counted by plating cells from swarms cultured on LB-1.5 agar. Cell density (CFU/mm^2^) was then calculated. The size of the colony and the cell numbers were determined by three independent experiments. Error bar represents standard deviation. Confocal micrographs of **(B)** *B. subtilis* F29-3(pSrf-gfp) and **(C)** *B. subtilis* 3610(pSrf-gfp) colonies that were respectively cultured on LB-0.4 and LB-0.7 agar plates (gray); the green color indicates the presence of GFP. Plates were inoculated with overnight cultures that were unconcentrated (1 ×), or log-phase cells that were concentrated 10-fold (10 ×). **(D)** LB-0.4 and **(E)** LB-0.7 agar plates were respectively inoculated with 10 μl 10-fold concentrated *B. subtilis* F29-3 or 100 μl unconcentrated *B. subtilis* 3610 overnight cultures. Bacteria were subsequently cultured for the time indicated. GFP and σ^A^ in the cell lysates were analyzed by immunoblotting. The amounts of σ^A^ were used as a loading control. A band larger than that of σ^A^ in strain F29-3 was nonspecifically detected by anti-σ^A^ antibody.

It is well known that transcription of the surfactin synthetase operon*, srfA*, is regulated by quorum sensing (Oslizlo et al., 2014). Our study showed that a mutant F29-3 strain (FK1829) with a mutation in *comA*, which encodes a key quorum sensing transcription factor (Roggiani and Dubnau, 1993; Tortosa et al., 2001; Comella and Grossman, 2005), did not swarm (Table 2), suggesting that quorum sensing is involved in F29-3 swarming. To further investigate this, we inoculated an LB-0.4 plate with 1 μl of F29-3 containing the plasmid pSrf-gfp, which contains a *srfA* promoter fused with a *gfp* reporter gene. We found that the size of the colony increased slowly during the first 4 h of culturing (Figure 8A) and then increased at a speed of about 1 mm/h. Meanwhile, cell density within a colony remained relatively constant during the first two h of culturing, at about 2 × 10^5^ CFU/mm^2^ (Figure 8A). However, densities began to increase after Hour 2, ultimately reaching a plateau of 4 × 10^6^ CFU/mm^2^ around Hours 5 and 6 (Figure 8A). Using a confocal laser-scanning microscope, we found that the colony began to exhibit green fluorescence at 4 h after inoculation (Figure 8Be). We also inoculated LB-0.4 agar plates with 10 μl of 10-fold concentrated F29-3(pSrf-gfp) cells, and found that the colony started to exhibit green fluorescence at 2 h after inoculation (Figure 8Bi); fluorescence became prominent after Hour 3 (Figures 8Bj–l). A similar study was conducted using 3610(pSrf-gfp) on 25-ml LB-0.7 agar. We found that when plates were inoculated with 10-fold concentrated cells according to the method described by Patrick and Kearns (2009), bacteria in the inoculum initially exhibited a high level of GFP expression, and these levels persisted throughout the 4-h post-inoculation period (Figures 8Cf–j). This indicates that the high bacterial density at the time of inoculation causes the strain to activate the *srfA* promoter and transcribe *gfp* at high levels. However, when LB-0.7 plates were inoculated with 3610 cells that had not been concentrated, the colony exhibited only small amounts of green fluorescence at the colony edges during Hour 0 and Hour 1 post-inoculation, as bacterial densities are higher at the edges (Figures 8Ca,b). At Hour 2, strong green fluorescence was observed at the edge of the colony, with small amounts of fluorescence observed in the inner areas (Figure 8Cc); green fluorescence was strongly expressed overall after Hour 3 (Figures 8Cd,e). These results suggest that *B. subtilis* swarms start to transcribe the *srfA* operon after bacterial density increases, which leads to the synthesis of surfactin and subsequent expansion of bacterial colonies. We further conducted an immunoblot study to show that a quorum sensing response does occur in a swarm. We found that inoculating plates with unconcentrated F29-3 cultures did not yield enough cells and proteins to allow us to assess GFP expression levels. Therefore, we inoculated 10 μl of 10-fold concentrated F29-3 cells on LB-0.4 plates, and pooled cells from 30 plates to assess the expression levels of GFP at each time point. We found that F29-3 did not express GFP during the first 2 h of culturing, but began to express GFP at Hour 3. Meanwhile, the amount of σ^A^, which was used as a loading control, remained at a constant level (Figure 8D). In the immunoblot analysis of GFP in 3610 cultures, plates inoculated with 10-fold concentrated cells expressed GFP at a constant level over time, and bacterial density-dependent surfactin synthesis was not observed. This was likely because the quorum sensing response had already been induced at inoculation. Therefore, we further inoculated LB-0.7 plates with 100 μl of unconcentrated 3610 cells, and subsequently found that GFP levels were initially high during Hour 1 after inoculation, but dropped gradually at Hours 2 and 3 (Figure 8E, lanes 3, 4). The amount of GFP then increased, and at Hours 5 and 7, cells expressed GFP at a high level (Figure 8E, lanes 5, 6). Meanwhile, levels of σ^A^ remained relatively constant (Figure 8E), indicating that changes in GFP levels were not attributable to differences in the amount of proteins loaded to each lane. These results suggest that quorum sensing occurs in swarms, and subsequently induce the synthesis of surfactin to facilitate swarming.

## Discussion

Swarming is a fascinating phenomenon, in which microorganisms work collectively as one to migrate *en masse* on an agar surface. Among the microorganisms that swarm, undomesticated *B. subtilis* has received particular attention due to its unparalleled migration speeds (Kearns and Losick, 2003), and because many strains produce tendrils with fractal-like patterns (Julkowska et al., 2004; Hamze et al., 2009; James et al., 2009). Our study shows that F29-3 contains genes that are necessary for swarming (Tables 1, 2), including those involved in flagellar and surfactin biosynthesis. However, *B. subtilis* F29-3 swarm at a speed slower than 3610, and may form tendrils on LB agar (Figure 1Al). We found that the amount of surfactin produced by F29-3 in LB broth after 16 h of culturing is about 18,000-fold less than that produced by strain 3610 (Figure 2; Table 3). This may explain why F29-3 does not swarm as fast as 3610 on LB agar, and why small amounts of surfactin are sufficient to promote *B. subtilis* F29-3 swarming.

Microscopic and macroscopic examinations in this study reveal that colonies formed by F29-3 contain water (Figures 3, 4). We show that *B. subtilis* 3610 also forms colonies containing water, and not only on LB-0.5 agar as previously reported (Be'er and Harshey, 2011), but also on LB-0.7 agar (Figures 6, 7). We found that strain 3610 forms a water layer of only 14 μm in height, and this shallowness explains why the presence of water was not detected by high-magnification phase contrast microscopy of LB-0.7 agar. The presence of water in swarms formed by gram-negative bacteria has been well documented. Partridge and Harshey (2013) proposed a model that the flagellar motion of *Salmonella enterica* disturbs the agar surface and causes water to seep from agar, which has been put forth as a mechanism of water extraction utilized by bacteria. However, we found that the flagellar mutants of F29-3 and 3610 (FW463 and DS1677) incorporate water in their colonies, and this water was shown to flow out of colonies cultured on tilted plates (Figures 3Bd, 6Cd), suggesting that mechanisms other than flagellar motion may also be involved in *B. subtilis* water extraction. In the case of *E. coli*, the water in a swarm was found to flow back and forth between the edge and interior of a swarm (Wu and Berg, 2012), and the swarm also contains a narrow high osmolarity region at the edge, where lipopolysaccharides (LPS) may be utilized for water extraction (Ping et al., 2014). *B. subtilis* may also use osmolytes to extract water, but this organism does not produce LPS, and possible osmolytes have not been identified as yet.

Since our results show that F29-3 only produces small amounts of surfactin in LB medium (Figures 2A,B), we tested whether adding surfactin, a powerful surfactant, to culture medium changes the swarming behavior of F29-3. We found that adding surfactin not only accelerated the speed of F29-3 swarming, but also created a 14-μm-high water layer (Figure 5B) similar to that formed by 3610 (Figures 6B, 7). Furthermore, the bacteria form a thin colony (Figure 5Ac) similar to that observed for 3610 post-swarming (Figure 6Ca), thus demonstrating that surfactin levels can influence swarming behaviors in these two strains. In addition, surfactin, as well as NP-40 and Triton X-100, appears to promote F29-3 tendril formation (Figures 5Ab,d,Ca,b), suggesting that weakening colony water surface tension with surfactin can also induce the formation of tendrils.

Quorum sensing regulates transcription of the surfactin synthetase operon and the expression of surfactin (Oslizlo et al., 2014). Our study shows that an F29-3 strain with a mutation in *comA* (FK1829) is unable to swarm (Table 2). Since, ComA is a key quorum sensing transcription factor (Tortosa et al., 2001; Comella and Grossman, 2005), the results suggest that quorum sensing is involved in *B. subtilis* swarming. Earlier, studies showed that inoculating agar plates with concentrated cultures of 3610 shortens the swarm lag period and promotes swarming (Kearns and Losick, 2003), suggesting that quorum sensing plays an important role in swarming. Our confocal microscopy results further reveal that *gfp* transcribed from the *srfA* promoter is expressed post-inoculation after cell density increases (Figures 8A–C), and this was confirmed by measuring GFP expression levels via immunoblotting (Figures 8D,E). These results show that a quorum sensing response occurs in high-density bacterial colonies, which triggers the expression of surfactin and leads to swarming. Meanwhile, we notice that unlike F29-3, which has bacteria evenly distributed in a swarm during the first 6 h of culturing, 3610 has a dense population at the inoculation site with a thin layer of motile bacteria dispatched from the center and rapidly migrating toward the edge of the swarm (Figure 6Aa). The bacteria at the center may produce a large amount of surfactin to destroy the water surface tension to allow the water exacted from the agar to flow across the surface of the plate. We also observed that when an F29-3 colony is swarming at its maximum speed, usually between 5 and 12 h post-inoculation, the edge of the colony does not always expand evenly (Figure 3Ac). The uneven speeds at which migration and mergence take place at the front of a swarm may lead to the formation of holes in the colony, within which bacteria are absent (Figure 3Ac). In the case of 3610, uneven colony expansion with finger-shaped tendrils is also observed (Figure 6Aa), demonstrating that tendril formation and mergence are features commonly shared by the two strains. We have demonstrated that the tendrils formed by 3610 are actually water channels that contain migrating bacteria within (Figure 6A). Be'er and Harshey (2011) found that the surface of the 3610 colony is dynamic, supporting the fact that water in the channels is likely flowing on the agar surface.

In conclusion, we find that after inoculation, *B. subtilis* extracts water from the agar medium and multiplies. Surface tension of the water restricts bacteria from expanding their colony. As bacterial density increases, a quorum sensing response is triggered, leading to the production of surfactin. In strains such as F29-3, which produce low levels of surfactin, the water surface tension of colonies remains strong, causing bacteria to swarm at a slow speed. When the water surface tension at a particular location on the edge of a swarm is weakened, a stream of motile bacteria can flow out of the colony to form a tendril. In strains capable of producing large amounts of surfactin, such as 3610, the boundary caused by water surface tension of a swarm collapses, allowing water to flow out in water channels that contain bacteria swarming within, eventually to form a thin colony on the agar surface.

### Addendum

We find that different types of agar can influence swarming behavior. Both F29-3 and 3610 swarm faster on LB plates made of Difco agar, as compared to those containing Oxoid agar. The swarming toward gravity on tilted plates is observed only on Oxoid LB but not on Difco LB plates.

### Conflict of interest statement

The authors declare that the research was conducted in the absence of any commercial or financial relationships that could be construed as a potential conflict of interest.

## Supplementary material

Supplementary Figure 1 **Morphology of *B. subtilis* F29-3, FW463, and FK955 colonies**. Flagellar staining of strains F29-3 **(a)** and FW463 **(b). (c)** Water (1 μl) or **(d)** 1 μl of 1μg surfactin solution was applied to the center of LB-0.4 plates. After drying the plates for 20 min, 1μl of overnight FK955 culture was applied to the locations where water and surfactin were applied. The plates were incubated for 24 h.

Supplementary Movie 1 **Movement of *B. subtilis* F29-3**. *B. subtilis* F29-3 was cultured on 8.5-ml LB-0.4 agar for 3 h, and the colony was observed using a stereomicroscope. This movie shows that bacteria are moving in all directions. A layer of nonmotile bacteria is floating and covering most of the colony. The bacterial movement was recorded using a stereomicroscope (Olympus SZX10) at a magnification of 25×.

Supplementary Movie 2 **Swarming of *B. subtilis* F29-3 at 12 h after inoculation**. The movie shows that bacterial aggregates (BA) are moving in all directions in about the 1-mm region of the colony. The bacteria inside the colony are nonmotile. LNB, layer of nonmotile bacteria.

Supplementary Movie 3 ***B. subtilis* F29-3 rafts. *B. subtilis* F29-3 forms rafts during swarming**. The bacteria swarmed in the presence of surfactin in a thin water layer. The movie was taken using a phase contrast microscope (Zeiss Axioskop) at a magnification of 400×.
