# Supplementary figures and images for: Water surface tension modulates the swarming mechanics of Bacillus subtilis
Source: Front Microbiol. 2015 Sep 24;6:1017. doi: 10.3389/fmicb.2015.01017 (PMC4616241; doi:10.3389/fmicb.2015.01017)

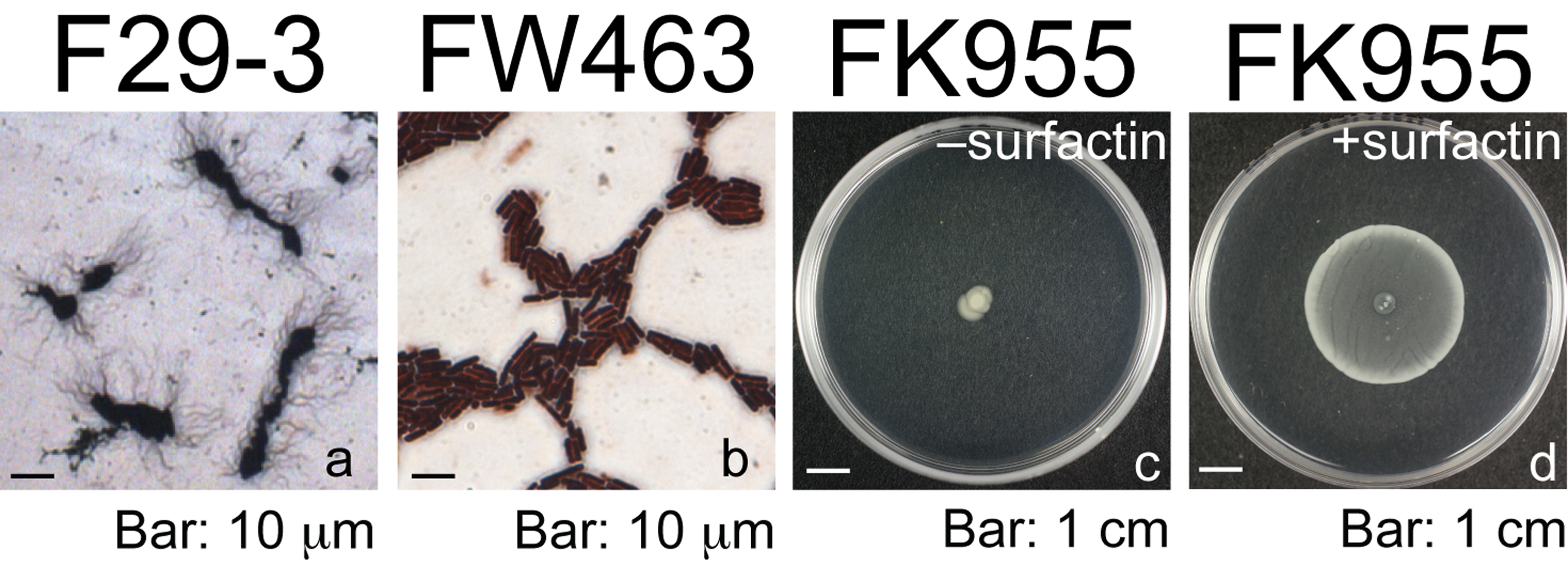

Supplement: Supplementary Figure 1 — Morphology of B. subtilis F29-3, FW463, and FK955 colonies. Flagellar staining of strains F29-3 (a) and FW463 (b). (c) Water (1 μl) or (d) 1 μl of 1μg surfactin solution was applied to the center of LB-0.4 plates. After drying the plates for 20 min, 1μl of overnight FK955 culture was applied to the locations where water and surfactin were applied. The plates were incubated for 24 h. [file Image1.TIF]
